# New Structural Insights into the Genome and Minor Capsid Proteins of BK Polyomavirus using Cryo-Electron Microscopy

**DOI:** 10.1016/j.str.2016.02.008

**Published:** 2016-04-05

**Authors:** Daniel L. Hurdiss, Ethan L. Morgan, Rebecca F. Thompson, Emma L. Prescott, Margarita M. Panou, Andrew Macdonald, Neil A. Ranson

**Affiliations:** 1 Astbury Centre for Structural Molecular Biology, University of Leeds, Leeds LS2 9JT, UK

## Abstract

BK polyomavirus is the causative agent of several diseases in transplant patients and the immunosuppressed. In order to better understand the structure and life cycle of BK, we produced infectious virions and VP1-only virus-like particles in cell culture, and determined their three-dimensional structures using cryo-electron microscopy (EM) and single-particle image processing. The resulting 7.6-Å resolution structure of BK and 9.1-Å resolution of the virus-like particles are the highest-resolution cryo-EM structures of any polyomavirus. These structures confirm that the architecture of the major structural protein components of these human polyomaviruses are similar to previous structures from other hosts, but give new insight into the location and role of the enigmatic minor structural proteins, VP2 and VP3. We also observe two shells of electron density, which we attribute to a structurally ordered part of the viral genome, and discrete contacts between this density and both VP1 and the minor capsid proteins.

## Highlights

• The first 3D structure of native, infectious, human BK polyomavirus
• Show the location and extent of the minor capsid proteins
• Identify contacts between all three structural proteins and the packaged genome
• Describe structural organization within the viral minichromosome

## Graphical Abstract

**Figure:**
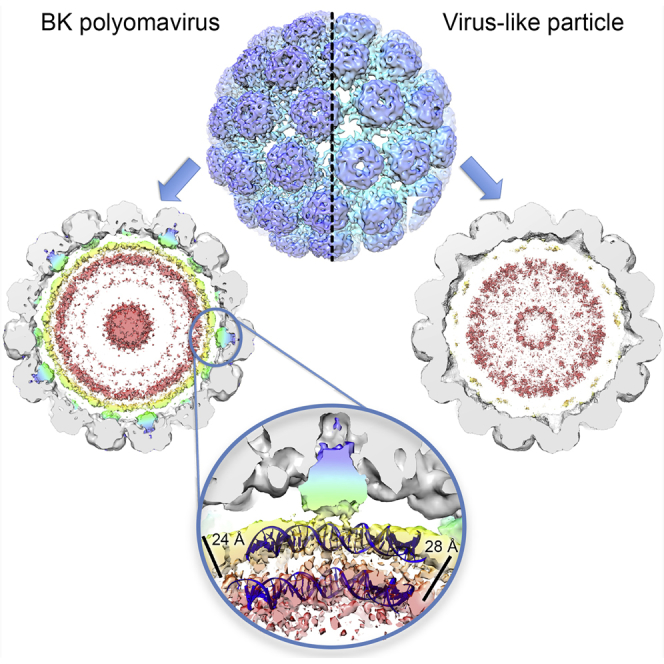

## Teaser

Hurdiss et al. present the first structure of human BK polyomavirus at 7.6 Å and compare it with a VP1-only VLP at similar resolution, providing new insights into the location of minor capsid proteins, genome recognition, and organization of the viral minichromosome.

## Introduction

Polyomaviruses are small, non-enveloped, double-stranded DNA (dsDNA) viruses belonging to the Polyomaviridae which use mammals, birds, and fish as their natural hosts (White et al., 2013, Peretti et al., 2015). The first two human polyomaviruses discovered, BK polyomavirus (BK) and JC polyomavirus (JC) were named after the index case patients upon their discovery more than 40 years ago (Padgett et al., 1971, Gardner et al., 1971). The last decade has seen the discovery of a number of new polyomaviruses linked to human disease, including trichodysplasia spinulosa-associated polyomavirus (van der Meijden et al., 2010) and Merkel cell polyomavirus (Feng et al., 2008), which cause skin lesions and an aggressive skin carcinoma, respectively. These discoveries have led to a resurgence of interest in polyomavirus biology (DeCaprio and Garcea, 2013).

BK is an opportunistic pathogen, capable of causing several diseases in the immunosuppressed (Knowles, 2006). Infection with BK typically occurs in childhood, and about 80% of adults have a persistent, lifelong infection in the kidney and urinary tract (Chesters et al., 1983). However, in the immunosuppressed, BK can become reactivated, resulting in shedding into the urine because of increased replication in the absence of competent immune surveillance and control (Ahsan and Shah, 2006). Such an increase in replication is ultimately linked with serious health problems, including polyomavirus-associated nephropathy (PVAN; Balba et al., 2013) and hemorrhagic cystitis (Dropulic and Jones, 2008) in patients who have received kidney and bone marrow transplants, respectively. For example, up to 10% of kidney transplant patients experience PVAN, and up to 90% of these will go on to lose their graft (Ramos et al., 2009). The incidence of BK-related disease is rising owing to the increasing number of transplants, and the immunosuppressive drug regimes used to support such patients (see review by Bennett et al., 2012). Generic antiviral drugs such as Cidofovir can be used, but have low efficacy and are themselves associated with nephrotoxicity (Safrin et al., 1997). No antiviral drugs that specifically target BK, or indeed any human polyomavirus, are currently available. Treatment is typically limited to a reduction in immunosuppression, which runs very real risks of transplant rejection (Kuypers, 2012, Vats et al., 2006). A better understanding of the polyomavirus life cycle in general, and BK in particular, is needed if we are to identify new targets for antiviral therapy. This may be particularly important in an age where the use of immunosuppressive drugs are becoming more widespread as treatment for a wide range of non-transplant patients (Araujo et al., 2011).

A detailed knowledge of structure is an essential prerequisite for efforts to understand the BK life cycle and treat associated diseases. High-resolution structural information for human polyomaviruses is currently lacking. Indeed, much of our current understanding of polyomavirus structure comes from the pioneering work of Caspar (Griffith et al., 1992, Rayment et al., 1982) and Harrison (Stehle et al., 1996, Stehle and Harrison, 1996) on the archetypal polyomaviruses SV40, and murine polyomavirus (MPV). The crystal structures of these viruses revealed that polyomavirus capsids consist of 360 copies of the major capsid protein VP1 (Liddington et al., 1991). These VP1 molecules form 72 pentameric structures, or pentons, that form the basic building block (or capsomere) of the capsid. Each penton consists of a ring of five β-barrel-containing VP1 monomers (Nilsson et al., 2005). Together these form a *T* = 7d lattice, with an invading arm comprised of the C termini of each VP1 undergoing exchange with neighboring pentons to stabilize the capsid shell. Structural studies have also highlighted the importance of calcium ions and disulfide bonds between pentons in capsid assembly (Ishizu et al., 2001). Although there are no X-ray structures for intact human polyomaviruses, the isolated pentons from both JC and BK have been studied and have shed much light on the binding of cell surface receptors (Neu et al., 2010, Neu et al., 2013). However, in all existing polyomavirus X-ray structures, the N-terminal 13–15 residues of the VP1 subunits were not crystallographically resolved, which was proposed to result from disorder in this region (Gillock et al., 1997). Subsequent biochemical studies demonstrated that these residues contain a DNA-binding domain (DBD; Li et al., 2001, Gillock et al., 1998). When expressed alone, VP1 is able to self-assemble into virus-like particles (VLPs) and package dsDNA molecules of a comparable size with the viral genome (Gillock et al., 1997, Gillock et al., 1998). It has therefore been proposed that this N-terminal DBD extends toward the center of the native particle to interact with the encapsidated ∼5.2 kB dsDNA genome (Liddington et al., 1991), but this has not been directly visualized.

The minor capsid proteins VP2 and VP3 are not resolved in existing X-ray crystallography structures. VP2/3 are expressed from the same open reading frame; however, VP2 has an extended N terminus, which contains a site for myristoylation (Krauzewicz et al., 1990). A single copy of VP2/3 has been proposed to bind within a cavity on the internal face of each VP1 penton. It has been hypothesized this occurs in one of five symmetry-related orientations, consistent with previous difference maps calculated from very-low-resolution X-ray diffraction data (Griffith et al., 1992). The binding of the common C-terminal 105 residues of VP2/3 in this location has been observed (Chen et al., 1998), although only ∼20 amino acids were crystallographically ordered and resolved at high resolution. This region of VP2/3 has been proposed to insert in a hairpin-like manner into the cavity of the VP1 penton, where it binds via hydrophobic interactions. However, much of the VP2/3 sequence is thought to be intrinsically disordered (Chen et al., 1998), with the common C-terminal region containing an additional DBD to that found in VP1. In SV40, VP2/3 are essential for viral morphogenesis, as shown by mutagenesis experiments (Clever et al., 1993, Dean et al., 1995). However, direct interactions between the minor capsid proteins with a packaged polyomavirus genome have not been visualized structurally. Both SV40 and BK VP2/3 proteins also contain a C-terminal nuclear localization signal (NLS). Mutations of basic residues in this region have been shown to decrease nuclear entry during host cell infection (Nakanishi et al., 2002, Nakanishi et al., 2007, Bennett et al., 2015).

Polyomavirus genomes are encapsidated with the cellular histone proteins H2A, H2B, H3, and H4 (Pagano, 1984), forming a chromatin-like structure termed a “minichromosome”; early work suggested it contains 20–24 nucleosomes (Cremisi et al., 1975, Griffith, 1975, Müller et al., 1978). However, unlike cellular chromatin, polyomavirus minichromosomes lack histone H1 (Fang et al., 2010), which is thought to be required for chromatin compaction (Thoma et al., 1979). This implies that the minichromosome is not highly compacted within the virus particle. Early attempts to capture the structure of isolated SV40 minichromosomes were carried out using cryo-electron microscopy (EM) (Dubochet et al., 1986). This revealed that the isolated genome adopts a range of structures reminiscent of cellular chromatin when outside the capsid, and that the structure observed varies depending on pH (Christiansen et al., 1977) and salt concentration (Griffith, 1975). However, no structural information for the minichromosome, in situ, has been reported, although small-angle X-ray scattering and coarse-grained computer simulations suggest that packaged nucleosomes lack a highly ordered structure, but are bound at the inside surface of the capsid layer (Saper et al., 2013).

In this study, we extend existing observations from a range of disparate systems and bring them together in the study of a single, pathogenic human polyomavirus. We present the first subnanometer-resolution solution structures of any polyomavirus, with the structure of native, infectious BK virion and of a BK VP1-only VLP. Together, these structures unambiguously identify the location of VP2/3 within the capsid of a human polyomavirus. We also identify discrete bridges of electron density, which connect the VP1 N terminus to packaged DNA, and observe points of contact between density for the minor capsid proteins and the genome. Furthermore, we see shells of density in the center of the virion that give the first structural evidence about how the minichromosome could be packaged.

## Results

### Production of Infectious BK Polyomavirus

**Figure 1:**
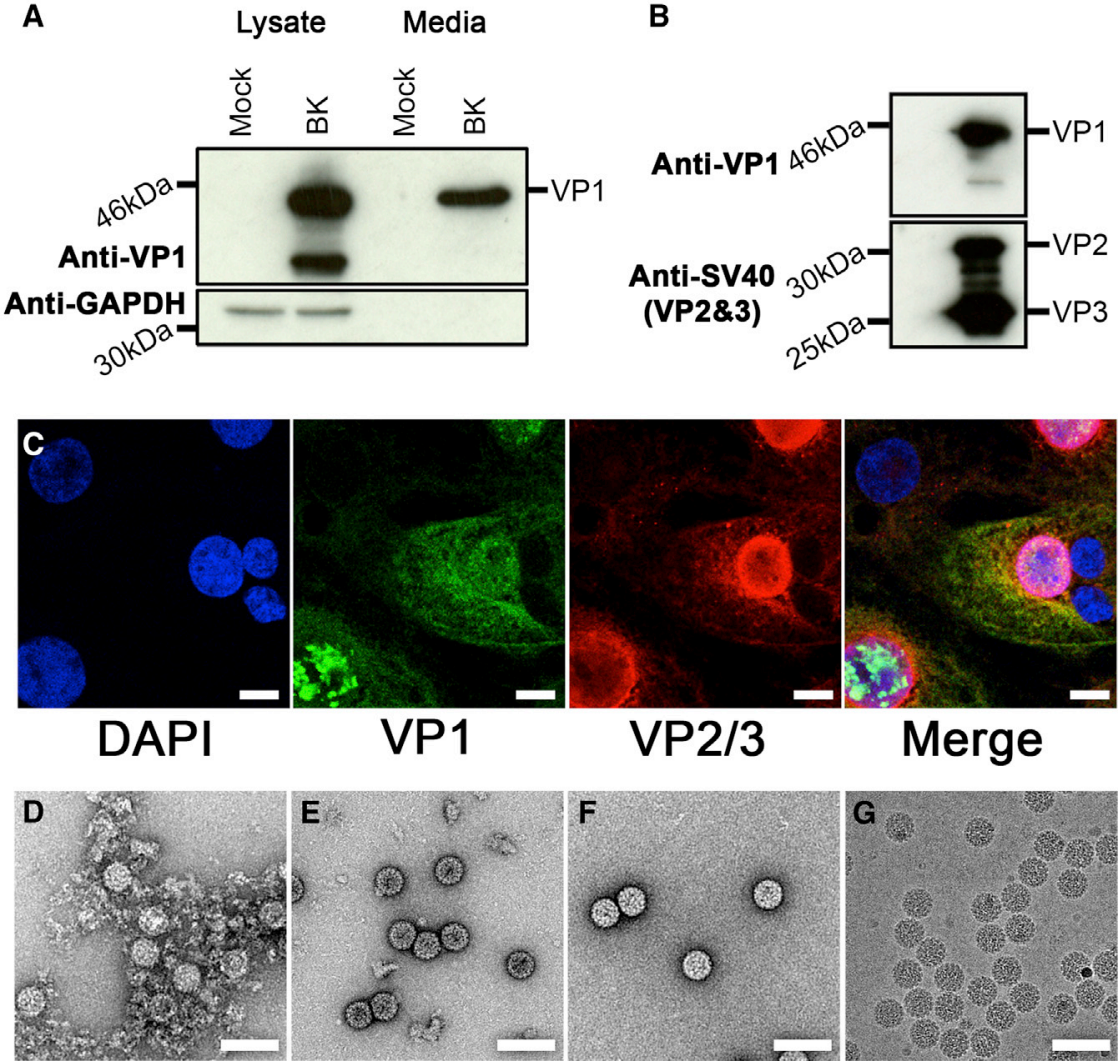
Purified Virions Are Infectious (A) Vero cell lysate and 30 μl of growth media were analyzed by western blotting to determine the presence of VP1 capsid protein and GAPDH expression. (B) The lysate from RPTE cells infected by gradient purified virions was separated by SDS-PAGE and analyzed by western blotting for the VP1 and VP2/VP3 capsid proteins. (C) RPTE cells were grown on glass coverslips and infected with virions; 3-dpi slides were fixed with 4% paraformaldehyde and stained with antibodies against VP1 (green) and VP2/VP3 (red). The nuclei were visualized with DAPI (blue). Scale bars, 10 μm. (D) Negative-stain electron micrograph of virions present in the Vero cell lysate. (E) Partially purified virions following centrifugation through a cesium chloride gradient. (F) Purified virions following final purification step through a centrifugal concentrator (right). (G) Purified virions suspended in a layer of vitreous ice following cryo-grid preparation. Scale bars, 100 nm.

To produce virus for structural studies, the circularized Dunlop strain genome of BK was transfected into Vero cells, a cell line derived from monkey kidney epithelial cells, which is ideal for culturing the virus. Ten days post transfection, cells were lysed, and the resulting crude cell lysate containing BK virions was used to infect naive Vero cells, which were grown for a further 14 days. Western blotting analysis showed that the major structural protein VP1 was present in both the media and crude cell lysate (Figure 1A), demonstrating that BK replicates in Vero cells, and suggesting that much of the virus made is not released into the media but remains inside cells. Virions were then purified as described previously (Jiang et al., 2009), by centrifugation through a cesium chloride gradient.

To confirm that this material was infectious, an aliquot of the purified virus was used to infect primary renal proximal tubular epithelial (RPTE) cells, which were analyzed 3 days post infection by western blotting using antibodies against VP1 and VP2/3. All three structural proteins are present in the virus particles (Figure 1B). The infected cells were also analyzed by quantitative PCR using primers against the BK genome, which gave a genome copy number of ∼1 × 10^8^ per μg of total cell-extracted DNA, verifying that the infection was productive as both genome replication and late gene expression had occurred. RPTE cells grown on glass coverslips were also analyzed by immunofluorescence microscopy 3 days post infection with antibodies against VP1 and VP2/VP3 (Figure 1C). While low-level diffuse staining for the capsid proteins can be observed throughout the cytoplasm, the structural proteins are primarily localized in the nucleus, with VP1 staining appearing as distinct puncta.

Negative-stain EM images (Figure 1D) confirm the presence of polyhedral particles with a diameter of 45–50 nm in the crude cell lysate, together with copious cellular debris. However similar images of the CsCl-gradient-purified virus showed that although considerably purer than the crude cell lysate, significant low-molecular-weight contaminants remained (Figure 1E). An additional purification step of centrifuging through a 1-MDa spin concentrator was therefore used. The virus, which was retained by the filter while contaminants washed through, was essentially pure after this step (Figure 1F) and taken forward for cryo-EM (Figure 1G).

**Figure 2:**
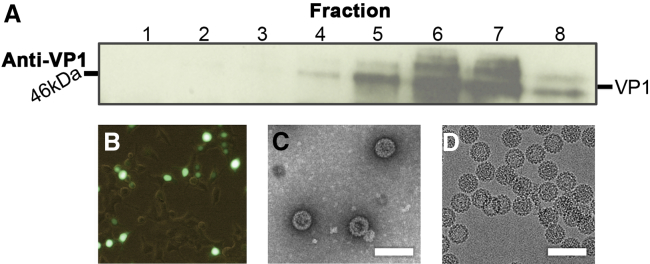
BK Polyomavirus VLP Production in HEK293TT Cells BK Polyomavirus VLPs produced in HEK293TT cells can package plasmid DNA and can be used as a gene delivery vector. (A) Western blot for VP1 expression in each fraction from the iodixonal gradient. 20 μl of the gradient solution was loaded and VP1 levels were detected using a VP1-specific antibody. (B) Purified VLPs were used to transduce HEK293TT, and the presence of EGFP was analyzed by light microscopy. The image is representative and approximately 65% of the cells were transduced. (C) Negative-stain micrograph of purified VLPs. (D) Cryo-electron micrograph of purified VLPs suspended in vitreous ice. Scale bars, 100 nm.

To produce VLPs for comparison with the native virion, HEK293TT cells were transfected using a codon-optimized VP1 expression plasmid, together with a reporter plasmid encoding EGFP. The resulting VLPs were purified on an OptiPrep gradient and the presence of VP1 detected via western blotting (Figure 2A). The purified VLPs were used to transduce naive HEK293TT cells, with the resulting fluorescence-positive cells demonstrating the VLP's ability to both package the EGFP reporter plasmid and deliver it across cell membranes (Figure 2B). For cryo-EM studies, VLPs were concentrated in the same manner as the virions. Negative-stain (Figure 2C) and cryo-EM images (Figure 2D) of VLPs demonstrated that they have a size and morphology indistinguishable from the native virion. We further characterized the virion and VLP by assessing their histone and DNA content (Figure S1). Each type of capsid packages some genomic DNA, but the virion contains the BK genome and more histones, while the VLP appears to package the high copy number EGFP reporter plasmid and a lower level of histone proteins. These observations are in agreement with previous studies which have demonstrated the ability of SV40 and MPV VP1 protein to promiscuously package cellular DNA and histones (Trilling and Axelrod, 1970, Gillock et al., 1997).

### The Solution Structure of Human BK Polyomavirus

**Figure 3:**
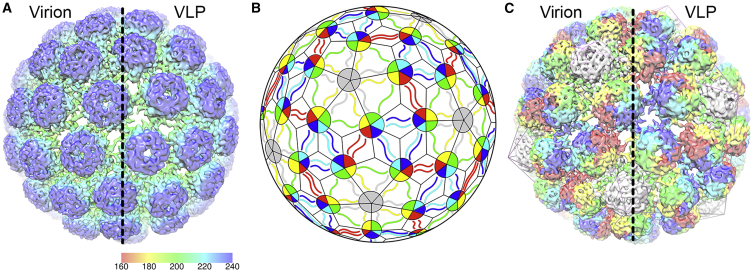
The Cryo-EM Structures of the BK Virion and VLP (A) An external view of the virion (left) and VLP (right) shown at contour levels of 0.022 and 0.009, respectively. The electron density maps have been sharpened using a negative B factor correction of (*B* = −456 and −804 Å^2^), and the density is colored using a radial scheme shown in Ångströms. (B) The architecture of a polyomavirus capsid showing how the *T* = 7d capsid is built from 72 pentamers of VP1, and an identical VP1 polypeptide sequence is found in six distinct quasi-equivalent conformations in the capsid shell (1, red; 2, yellow; 3, green; 4, cyan; 5, blue; and 6, gray). (C) The cryo-EM structure of the virion (left) and VLP (right) colored according to the scheme in (B), showing the arrangement of different VP1 molecules in three dimensions.

Images of unstained, frozen-hydrated virions and VLPs were recorded using an electron microscope with a direct electron-detecting camera, allowing us to determine the solution structures of the virion to 7.6 Å and VLP to 9.1 Å (Figure 3A). These are the two highest-resolution EM structures for any polyomavirus capsid to date (Li et al., 2003, Nilsson et al., 2005, Shen et al., 2011, Li et al., 2015) and allow us to visualize secondary structural elements (α helices and β sheets, although not individual β strands). Both the native virion and VLP are isometric particles with a diameter of ∼500 Å, similar to that described previously for other polyomaviruses, including SV40 (Stehle et al., 1996, Liddington et al., 1991) and MPV (Stehle and Harrison, 1996). The capsids have a *T* = 7d quasi-symmetry; and are built from 72 pentameric capsomers, which are easily visible in the maps. Each penton contains five copies of the major structural protein VP1, and there are six distinct conformations of VP1 in the shell (Figure 3B). The pentamers are tied together using C-terminal arms, with each pentamer of VP1 donating and receiving five such arms to/from adjacent pentamers (Figure 3C). A homology model of the BK VP1 asymmetric unit was generated using the SWISS-MODEL server and flexibly fitted into the corresponding density of the virion map. A homology model of the BK VP1 asymmetric unit was generated based on SV40 using the SWISS-MODEL server and flexibly fitted into the corresponding density of the virion map. At the resolution presented, the gross fold of BK VP1 is extremely similar to previous structures of the non-human polyomavirus, SV40, which shares 81.7% sequence identity in the major structural protein.

### Minor Capsid Proteins and the Organization of the dsDNA Genome

**Figure 4:**
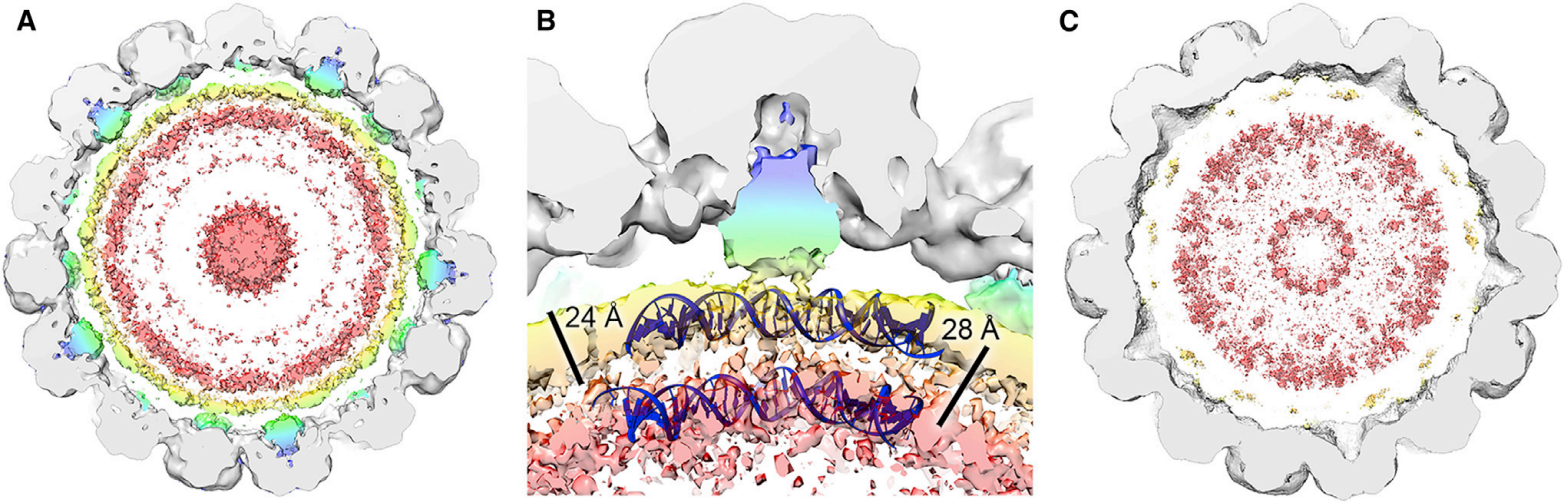
Minor Capsid Proteins and Genome Organization (A) A 40-Å thick slab through the unsharpened/unmasked virion map shown at a contour level of 0.0034. Pyramidal density below each VP1 penton and two shells of electron density adjacent to the inner capsid layer can be seen. The density within 6 Å of the fitted coordinates for SV40 VP1 is colored gray. The remaining density is colored in a radial color scheme. Density for VP2 and VP3 is colored blue→green, and for packaged dsDNA yellow→pink. (B) Enlarged view of the pyramidal density beneath a single VP1 penton of the virion shown at a contour level of 0.0032. Strands of dsDNA wrapped around a human histone octamer (PDB: 1AOI) are shown, indicating that the two shells of density have a comparable spacing. Discrete connective density between the pyramidal density and internal shells is also apparent. Scale bars shown. (C) A 40-Å thick slab through the unsharpened/unmasked VLP map shown at a contour level of 0.0011 which shows that density for the minor capsid proteins and two shells of electron density is absent.

Unlike previously described X-ray structures, our virion map contains significant density that we attribute to the minor capsid proteins VP2 and VP3 (Figure 4A). Low-resolution data from related viruses have suggested that VP2 and VP3 bind to the inner surface of a VP1 penton, but this has not been previously visualized in BK. This density is weaker (and at a lower resolution) than the VP1 shell, but can still be clearly seen even in a map which has been subjected to the B-factor correction used to reveal high-resolution features, the unsharpened/unmasked maps for the virion and VLP (9.1 and 11.07 Å, respectively) are deposited under the same EMDB codes as their refined counterparts. A depression on the inside surface of the VP1 penton appears to be completely filled with a conical density, and we attribute this to VP2 and/or VP3 on the basis that this is missing in the VLP structure (Figure 4B).

Both the virion and the VLP have additional density inside the capsid shell, which we propose to arise from the packaged dsDNA genome and EGFP reporter plasmid/cellular DNA, respectively. The virion map has strong density in two distinct shells directly beneath the capsid layer. The outermost of these is connected to the proposed VP2/3 density and the two shells have a thickness and radial spacing consistent with dsDNA wrapped around a histone octamer (Figure 4B) (Luger et al., 1997). The VLP map lacks these two discrete shells, and overall the density is weaker and more diffuse (Figure 4C).

**Figure 5:**
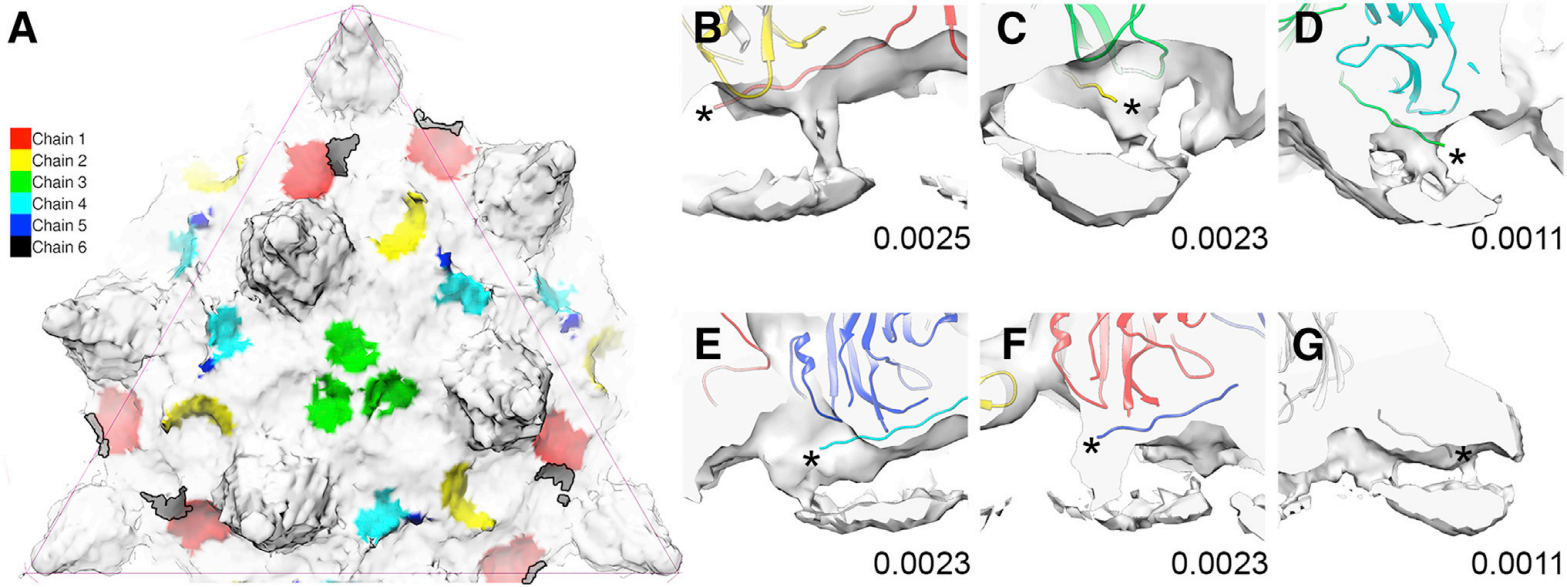
Visualization of Interactions between VP1 N Termini and Encapsidated Genome (A) The location of VP1 N termini binding to the viral genome relative to the minor capsid proteins, viewed down the capsid threefold axis of symmetry (chain color key shown). For visual clarity, density within 6 Å of the fitted coordinates for SV40 VP1 has been removed. A single asymmetric unit from the SV40 atomic model, colored according to the schematic shown in Figure 3B, was fitted as a rigid body into the unsharpened/unmasked virion map. The map was then segmented within 15 Å of the fitted model. (B–G) 20-Å thick slabs of density through the segmented map showing connective bridges of density between the capsid and packaged genome. Each of these is located beneath the N termini of each of the six VP1 quasi-equivalent conformers, which are denoted by asterisks (map contour levels shown).

In the virion map, we also see discrete bridges of density between the VP1 capsid and encapsidated dsDNA, the location of these relative to the virus capsid and minor capsid proteins is shown in Figure 5A. These bridges are situated beneath the N termini of each of the six VP1 quasi-equivalent conformers using the nomenclature adopted by Stehle et al. (1996) (Figures 5B–5G). Interestingly, the density associated with the N termini of chains 3 and 6 appear to be a weaker feature within the virion map. No equivalent features are visible in the VLP map. We propose that these densities correspond to some portion of the 13–15 N-terminal residues of VP1, which are not resolved in previous polyomavirus structures.

## Discussion

The structure of the wild-type, infectious BK virion presented here provides new insights into polyomavirus biology. The structure and arrangement of BK VP1 is remarkably similar to that of SV40, with minor rearrangements that do not affect either the gross fold of VP1, the size of the penton body, or the resulting icosahedral particle. The icosahedral averaging applied to the virion structure during refinement means that information for the VP2 and VP3 proteins is compromised. These are asymmetric proteins bound into a pentameric capsomer, presumably at one of five redundant binding sites. This capsomer then sits at both hexavalent and pentavalent positions within the *T* = 7d lattice. Thus, while the VP1 component of the structure is appropriately averaged, VP2 and VP3 (and their modes of binding) are incorrectly averaged, and the density observed is hard to interpret. However, some new conclusions can be drawn. The volume of the cone-shaped density corresponding to VP2/3 is 15–18,000 Å^3^ (at the contour shown in Figure 4B). Although the errors in such calculations can be considerable, these volumes are consistent with ∼32%–38% of a VP2 and 45%–55% of a VP3 molecule being present within the density shown (VP2 = 38.3 kDa; VP3 = 26.7 kDa, density of protein = 1.37 g/cm^3^). We also see discrete bridges from this VP2/3 density to an inner shell that we ascribe to a mixture of the remainder of VP2/3, the encapsidated dsDNA genome, and packaged histone proteins. There is little evidence to suggest that the packaged genome is icosahedrally ordered, meaning that the genome information is also obscured as a result of the symmetry imposed during image processing. There is also absolutely no suggestion from classification of the image data that the packaged chromosome is condensed into a solenoid-type structure within the virion, regardless of whether such structures may be possible for isolated minichromosomes (Dubochet et al., 1986). This is consistent with the absence of histone H1 from the virus. Despite the uncertainty caused by icosahedral averaging, the radial distribution of the density should be accurate, as seen in the encapsidated genomes from other dsDNA viruses (Lander et al., 2013, Jiang et al., 2006, Cerritelli et al., 1997). Strong density is observed inside the virion, as two ∼24-Å thick radial shells separated by a gap of ∼28 Å. The spacing of the radial density shells in the virion structure closely matches the spacing of dsDNA present within a human nucleosome (PDB: 1EQZ) (Luger et al., 1997). We do not see clear density for the histones themselves, although at lower contour levels the two layers begin to merge, presumably because of the contribution of density from disordered histone proteins. This is consistent with observations on other histone-containing complexes, such as a retroviral intasome complex (Maskell et al., 2015), where the ordered polyphosphate backbone of dsDNA is a particularly strong feature in density maps. These observations are consistent with simulations performed on SV40, which indicated that nucleosomes within the capsid center lack orientational order, while those in the layer adjacent to the capsid wall align with the boundary (Saper et al., 2013). Any structural information from the relatively disordered nucleosomes within the capsid center appears to be averaged out completely.

Although these are the first observations of genome packaging in a polyomavirus, density for genomic material has been observed in a number of other viruses by cryo-EM (e.g., Hesketh et al., 2015). For single-stranded RNA (ssRNA) viruses including *Heterocapsa circularisquama* RNA virus (HcRNAV; Miller et al., 2011) and Turnip crinkle virus (Bakker et al., 2012), a similar double-shelled pattern has been observed. This probably reflects the base-pairing of ssRNA to form a collapsed, partly double-stranded substrate for packaging, and the effect of basic RNA-binding domains (or “arms”) from the viral coat proteins in condensing the DNA. For a dsDNA virus, whose genome is much stiffer than a partly base-paired RNA, the outcome of packaging appears to be similar, with multiple shells of dsDNA packaged into many viruses albeit with the help of powerful packaging motors (Jiang et al., 2006, Lander et al., 2006). The density observed in the BK virion again has a similar size and spacing despite the lack of any packaging motor. Perhaps the role of histones in genome packaging for small dsDNA viruses is to exploit the host's own strategy for DNA compaction, overcoming the need for a packaging motor.

Unlike previous polyomavirus structures, we observe discrete bridges of density connecting the encapsidated dsDNA and VP1 in the virion map. This density is located beneath the N termini of the fitted SV40 crystal structure, indicating that it corresponds to the 13–15 residues not resolved in previous X-ray structures. Although the DNA-binding properties of the VP1 N terminus have been described biochemically, this is, to our knowledge, the first visualization of direct interaction between a polyomavirus capsid and its packaged genome, and suggests that ongoing structural work may be able to resolve these interactions at high resolution. These bridges of density suggest a role in genome packaging/recognition and/or capsid assembly, but the N termini may also facilitate genome targeting to the nucleus. Presumably this would be during viral uncoating, when the NLSs contained in these sequences become exposed.

The structures presented here are the first subnanometer-resolution structures of a native human polyomavirus, and give new insight into the organization of the minor capsid proteins, their interaction with packaged genome, and the organization of that genome within the native particle. It therefore provides the platform for future efforts to image the particle at higher resolution and without symmetry averaging as it goes through its life cycle.

## Experimental Procedures

### Cell Culture

Vero cells were maintained in DMEM with 10% fetal bovine serum (FBS) and 50 U/ml penicillin and streptomycin. Primary RPTE cells (Lonza) were maintained in renal epithelial growth medium with the REGM BulletKit supplements (Lonza). 293TT cells (Buck et al., 2004) were cultured in DMEM with 10% FBS and non-essential amino acids (Life Technologies). HEK293TT cells, a kind gift from Dr. Chris Buck (National Cancer Institute) were cultured in DMEM with 10% FBS and non-essential amino acids.

### Virus Growth

The BK Dunlop genome (a kind gift from Professor Michael Imperiale, University of Michigan) was excised from the pGEM7 Dunlop plasmid by *Bam*HI (New England Biolabs) digestion and then recircularized using T4 Ligase (New England Biolabs). Vero cells were seeded into T75 flasks and transfected with 4 μg DNA using NanoJuice (Novagen) according to the manufacturer's instructions with a DNA to Core ratio of 1:2 and DNA to Booster ratio of 1:3. Transfection complexes were removed 16 hr post transfection and replaced with fresh growth media. Ten days post transfection the cells were harvested by scraping into the media and subjected to three rounds of freeze-thaw using liquid N_2_. The crude virus stocks were used to infect flasks of 70% confluent Vero cells at 37°C; after 2 hr the virus was removed and replaced with fresh growth media.

### Virus Purification

Virions were harvested 14 days post infection and purified as described previously (Jiang et al., 2009). Briefly the cells were harvested by scraping and pelleted by centrifugation at 8,000 × *g*, the pellet was resuspended in buffer A (10 mM HEPES [pH 7.9], 1 mM CaCl_2_, 1 mM MgCl_2_, 5 mM KCl), sonicated in a water bath for 5 min and treated with 1 U/ml neuraminidase (Sigma) for 1 hr at room temperature, after having the pH adjusted to 6.0. The pH was adjusted back to 7.4 and the lysate heated at 40°C for 5 min. The lysate was pelleted at 16,000 × *g* for 5 min and then the pellet resuspended in buffer A and incubated with 0.1% (w/v) deoxycholic acid for 15 min before being pelleted again. The supernatants were combined over a 4-ml 20% (w/v) sucrose cushion in buffer A before centrifugation at 85,000 × g for 3 hr at 4°C in a Beckman SW28Ti rotor. The pellet was resuspended in 1 ml of buffer A and layered over a 6-ml preformed 1.2–1.4 g/cm^3^ CsCl gradient before centrifugation at 155,000 × *g* for 16 hr at 15°C in a Beckman SW40Ti rotor. The band containing mature virions was collected using a 26-guage needle and dialyzed against buffer A overnight at 4°C. For structural analysis by cryo-EM, virions were concentrated 20-fold in a centrifugal concentrator with a 1-MDa cut-off at 4°C for 15 min (Vivaspin 500, Vivaproducts).

### VLP Expression and Purification

VLPs were generated using an updated and slightly modified protocol that has been previously described (Touzé et al., 2001, Buck et al., 2004). Briefly, 293TT cells were co-transfected with a codon-modified BK polyomavirus expression plasmid for VP1 (pIaw), together with a reporter plasmid encoding EGFP. Forty-eight hours after transfection, VLPs were harvested and resuspended at a concentration of >100 million/ml in buffer A. Neuraminidase V (Sigma) was added to a final concentration of 1 U/ml, and the cell suspension was incubated for 15 min at 37°C, followed by cell lysis by the addition of 0.5% Triton X-100 (Sigma) and further incubation at 37°C for an additional 15 min. The lysate was then treated with 0.1% benzonase (Sigma) and 0.1% DNase (Sigma). Capsid maturation was allowed to proceed overnight at 37°C. Lysates were clarified by centrifugation at 5,000 × *g* for 10 min and purified through a 27% to 33% to 39% iodixanol gradient (Optiprep; Sigma). After purification, fractions were collected by gravity flow and a 10-μl sample was analyzed for VP1 expression by western blot. To determine the infectivity of the BK VLPs, HEK293TT cells were seeded at 150,000 cells per well in six-well plates, 24 hr prior to infection. 5 μg of purified BK VLPs (approximately 100× more than reported previously Schowalter and Buck, 2013) was added to the wells and incubated overnight. Cells were then analyzed by microscopy for EGFP expression. For structural analysis by cryo-EM, VLPs were concentrated 20-fold in a centrifugal concentrator with a 1-MDa cut-off at 4°C for 15 min (Vivaspin 500, Vivaproducts).

### Immunofluorescence

RPTE cells were grown on glass coverslips and infected at around 50% confluency with 100 genomes/cell of purified virus in opti-MEM. The cells were fixed 3 days post infection with 4% paraformaldehyde in PBS for 10 min, followed by permeabilization with 0.1% Triton in PBS for 5 min. The cells were stained with mouse anti-VP1 P5G6 (a gift from Professor Denise Galloway, Fred Hutchinson Cancer Research Center; used 1:500) and rabbit anti-SV40 VP2 + VP3 (Abcam; used 1:250), followed by Alexa Fluor 488 chicken anti-mouse and Alexa Fluor 594 chicken anti-rabbit (Life Technologies; used 1:500). ProLong Gold antifade mountant with DAPI (Life Technologies) was used to mount the coverslips, which were imaged using a Zeiss LSM700 inverted confocal microscope.

### Western Blotting

Triton lysis buffer (10 mM Tris [pH 7.6], 10 mM sodium phosphate, 130 mM NaCl, 1% Triton X-100, 20 mM *N*-ethylmaleimide, complete protease inhibitor cocktail; Roche) was used to harvest total cellular protein from the infected cells. 30 μg of lysate was separated by SDS-PAGE and following transfer to nitrocellulose was probed with the following antibodies diluted in 5% non-fat dried milk in TBS with 0.1% Tween 20: mouse anti-VP1 p5G6 (a gift from Professor Denise Galloway, Fred Hutchinson Cancer Research Center; used 1:5,000), rabbit anti-SV40 VP2 + VP3 (Abcam; ab53983; used 1:1,000), and mouse anti-GAPDH (Santa Cruz; used 1:5,000).

Protein concentrations for the VLPs were calculated using a bicinchonic acid assay according to the manufacturer's instructions (Pierce). Proteins from cell lysates were separated on 10% SDS-PAGE gels, with 20 μg protein loaded per sample. Proteins were then transferred by a semi-dry transfer method (Trans Blot SD Semi-Dry Transfer cell; Bio-Rad) onto a nitrocellulose membrane (GE Healthcare). Membranes were blocked with 5% milk solution and the following primary antibodies were used at a dilution of 1:5,000: VP1 P5G6 and GAPDH as a loading control. Horseradish peroxidase-conjugated mouse or rabbit secondary antibodies (Sigma) were used at a 1:5,000 dilution. Proteins were detected using WesternBright ECL (Advansta) and visualized on X-ray film.

### Quantitative PCR

Total DNA was extracted from the infected cells using the E.Z.N.A. Tissue DNA kit (Omega Bio-Tek) and 10 ng DNA was analyzed by quantitative PCR using the QuantiFast SYBR Green PCR kit (Qiagen) with the following primers against BKPyV Dunlop: BK Dunlop copy forward, TGT GAT TGG GAT TCA GTG CT; BK Dunlop copy reverse, AAG GAA AGG CTG GAT TCT GA. A serial dilution of the pGEM7 Dunlop plasmid was used to calculate the copy number per microgram of total DNA.

### Electron Microscopy

For negative staining, 3-μl aliquots of native, wild-type BK or VLP in buffer A were applied to continuous carbon grids that had been glow discharged for ∼30 s in air. The virus was then stained with 1% uranyl acetate solution and allowed to dry in air for ∼5 min. Samples were imaged on a Tecnai G^2^-Spirit transmission EM at 120 keV, equipped with a Gatan US1000XP CCD camera.

For cryo-EM, 3-μl aliquots of native, wild-type BK or VLP in buffer A (supplemented with 50 mM CaCl_2_ for VLP) were applied to Quantifoil R2/1 200-mesh Cu EM grids that had been glow discharged in air for ∼30 s. The samples were then blotted in 100% relative humidity and vitrified by plunging into liquid-nitrogen-cooled liquid ethane, using an FEI Vitrobot mark IV or Leica-EM-GP. Wild-type BK was imaged using an FEI Tecnai-F20 transmission electron microscope at 200 kV with a Gatan 626 holder and a Gatan K2-Summit direct electron detector. Images were recorded under low-dose conditions using the K2 camera in counting mode. Images were recorded at 19,000× magnification, at a dose rate of ∼4 e^−^/Å^2^/s, at 4 frames per second frame rate, and a 10-s exposure. The recorded images had a calibrated object sampling of 1.92 Å/pixel and a total accumulated dose of ∼40 e^−^/Å^2^. For VLP samples, grids were imaged at OPIC on an FEI Polara microscope. Images were obtained on a Gatan K2 summit camera operating in super-resolution mode, with a final pixel sampling 0.675 Å/pix.

### Image Processing

For the virions, a total of 432 micrographs were recorded, each consisting of a movie of 40 frames. Images were converted from Gatan DM4 format to MRC using the dm2mrc tool in IMOD (Kremer et al., 1996). A drift correction calculation was then performed on each stack of movie frames using Motioncorr (Li et al., 2013), writing out a drift-corrected average of frames of 5–25. This was done to eliminate both the earliest frames (where beam-induced movement is strongest), and the last frames (where radiation damage predominates). The defocus of each drift-corrected average was then determined using CTFFFIND3 (Mindell and Grigorieff, 2003), and was in the range 0.6–4.8 μm. All further image processing steps were performed in RELION (Scheres, 2012). Particles were picked manually and yielded a total dataset of 7,697 virions. Using iterative 3D classification into two classes, a subset of 2,237 images was taken forward for 3D reconstruction. The resulting map was sharpened using a B factor of −456 Å^2^ to give a final resolution of 7.6 Å using a gold-standard Fourier shell correlation between two independently refined halves of the dataset at 0.143. All figures were made using UCSF Chimera (Pettersen et al., 2004).

For VLPs, a total of 170 micrographs were recorded, each consisting of a movie of 40 frames. A drift correction calculation was then performed on each stack of movie frames using Motioncorr. The defocus of each drift-corrected micrograph was determined using CTFFIND3. All further image-processing steps were performed in RELION. Particles were interactively selected from all micrographs and yielded a total dataset of 2,888 particles. Using iterative 3D classification into two classes, a subset of 2,517 images was taken forward for 3D reconstruction. The resulting map was sharpened using a B factor of −804 Å^2^ and using the gold-standard Fourier shell correlation between two independently refined halves of the dataset at 0.143, the final resolution was 9.1 Å. All figures were made using UCSF Chimera (Pettersen et al., 2004).

### Homology Modeling and Flexible Fitting of the BK VP1 Structure

A homology model of the BKPyV VP1 asymmetric unit based on the crystal structure of SV40 (PDB: 1SVA) was built using the SWISS-MODEL server. This was then fitted (as a rigid body) into a corresponding segment of the BKPyV cryo-EM density map generated using UCSF Chimera. Flexible fitting of the homology model was then carried out using MDFF (Trabuco et al., 2008).

### Structure Deposition

The refined and unrefined maps of the virion and VLP reconstructions were deposited in the Electron Microscopy Data Bank (EMDB: 3283 and 3284), respectively. The coordinates for the VP1 homology model structure docked into the virion EM density were deposited in the PDB (PDB: 5FUA).

## Author Contributions

Conceptualization, D.L.H., E.L.M., A.M., and N.A.R.; Methodology, D.L.H., E.L.M., A.M., and N.A.R.; Investigation, D.L.H., E.L.M., R.F.T, E.L.P., and M.M.P.; Writing — Original Draft, D.L.H., A.M., and N.A.R.; Writing — Review & Editing, D.L.H., E.L.M, R.F.T., A.M., and N.A.R.; Visualization, D.L.H. and N.A.R.; Supervision, A.M. and N.A.R.

## Supplemental Information

Document S1. Supplemental Experimental Procedures and Figure S1

Document S2. Article plus Supplemental Information
