# Supplementary material for: New Structural Insights into the Genome and Minor Capsid Proteins of BK Polyomavirus using Cryo-Electron Microscopy
Source: Structure. 2016 Apr 5;24(4):528–36. doi: 10.1016/j.str.2016.02.008 (PMC4826271; doi:10.1016/j.str.2016.02.008)
Supplement: Document S1. Supplemental Experimental Procedures and Figure S1 [file mmc1.pdf]

Structure, Volume *24*

Supplemental Information

New Structural Insights into the Genome and Minor Capsid Proteins of BK Polyomavirus using Cryo-Electron Microscopy

Daniel L. Hurdiss, Ethan L. Morgan, Rebecca F. Thompson, Emma L. Prescott, Margarita M. Panou, Andrew Macdonald, and Neil A. Ranson

Supplemental Figures

Figure S1, related to Figures 1 and 2

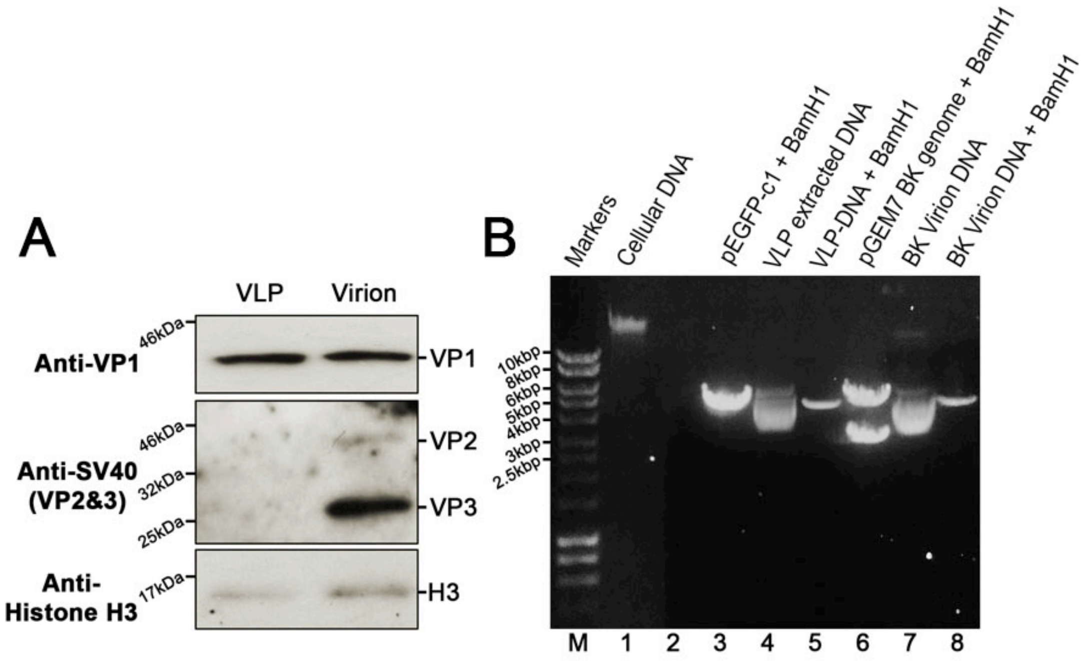

Supplemental Figure Legends

Figure S1, related to Figures 1 and 2

VLPs and virions contain cellular histones and DNA (a) Purified VLPs and virions were boiled in gel loading buffer and analysed by western blot for histone content using an anti-histone H3 antibody VP1 expression was used as a loading control and VP2/3 expression was used to confirm minor capsid protein expression in the virions alone. (b) BamHI digested and undigested DNA extracted from VLPs and virions was analysed on a 0.7% agarose gel. BamHI linearises the pEGFP reporter plasmid/viral genome and digests cellular DNA, shown by the presence of a single discrete band in the digested samples. Linearised pEGFP and pGEM7 BK genome were included as controls.

Supplemental Experimental Procedures

Analysis of VLP and virion histone content

Purified VLPs and virions were boiled in gel loading buffer and analysed by western blot (See-Experimental procedures: Western Blotting) for histone packaging using an anti-histone H3 antibody #3638 (Cell Signalling Technologies).

DNA extraction from VLPs and Virions

For analysis of packaged DNA in VLPs and virions, 50 μL of purified VLPs or virions were incubated at 56 °C in water containing 5 μL each of Proteinase K, 10% SDS and 0.5 M EDTA. After incubation, half of the sample was digested with 1 μL BamHI at 37 °C for 2 hours to allow analysis of packaged genome or plasmid DNA and cellular DNA. 5 μL of digested sample was then added to gel loading dye and loaded on to a 0.7% agarose gel for DNA analysis – see Agarose gel electrophoresis (below). As a control for cellular DNA, 100 ng of DNA extracted from HEK293TT cells using E.Z.N.A.® Tissue DNA Kit (Omega Bio-Tek) was loaded on the gel.

Agarose gel electrophoresis

Individual DNA samples (5 μl) were combined with 10 μl of 5 x DNA loading buffer and loaded into wells on a 0.7 % agarose gel. HyperLadderTM 1 Kb - Bioline (6 μl) was also loaded into one of wells to allow for easy size determination of DNA bands. The samples were run in a Mini- Sub® Cell GT (Bio-Rad). These were run at 80 Volts for 60 minutes in Tris- acetate-EDTA (TAE) buffer. A 10 μl aliquot of stock SYBR® Safe DNA Gel Stain (InvitrogenTM) was added to the TEA buffer.
